# Maternal and Perinatal Outcomes in Oligohydramnios: A Cross-Sectional Analysis of Pregnancies Between 28 to 42 Weeks of Gestation

**DOI:** 10.7759/cureus.79232

**Published:** 2025-02-18

**Authors:** Akash A Sawant, Sachin Wankhede, Sarika Thakare, Gaurang N Narayan, Indrakshi Saha, Amruta Vernekar, Bhagyashree D Wagaskar, Tejaswini S Khandare, Vidya S Hatwar, Rajlaxmi L Deshmukh

**Affiliations:** 1 Obstetrics and Gynecology, Indira Gandhi Government Medical College and Hospital, Nagpur, IND; 2 Obstetrics and Gynaecology, Indira Gandhi Government Medical College and Hospital, Nagpur, IND; 3 Obstetrics and Gynaecology, Squad Medicine and Research, Tiruchirappalli, IND; 4 Obstetrics and Gynaecology, Mahatma Gandhi Mission (MGM) Medical College and Hospital, Nagpur, IND

**Keywords:** feto-maternal outcome, high-risk obstetrics, maternal-fetal medicine, oligohydramnios, perinatal outcome

## Abstract

Background

Oligohydramnios, defined as an amniotic fluid index (AFI) <5 cm or a single deepest pocket <2 cm, is associated with increased risks of intrauterine growth restriction (IUGR), umbilical cord compression, and perinatal morbidity. Despite extensive research, controversy remains regarding the optimal management strategy for pregnancies complicated by oligohydramnios. This study evaluates maternal and perinatal outcomes in oligohydramnios to guide clinical decision-making.

Methods

A hospital-based cross-sectional study was conducted over two years (October 2022 to September 2024) at a tertiary care center. The study included 188 pregnant women between 28 and 42 weeks of gestation diagnosed with oligohydramnios. Data on maternal demographic characteristics, pregnancy complications, fetal monitoring parameters, mode of delivery, and neonatal outcomes were collected and analyzed using chi-square and t-tests, with a significance level of p<0.05.

Results

The study revealed significant maternal and perinatal complications in oligohydramnios. The mean maternal age was 25.4 ± 4.2 years, with 61.7% primigravida. Cesarean section was the predominant mode of delivery (69.1%) due to fetal distress. Low birth weight (<2.5 kg) occurred in 53.19% of neonates, with 29.73% having an Apgar score ≤7 at one minute. Neonatal intensive care unit (NICU) admission was required in 14.36% of cases, and perinatal mortality was 8.5%. Non-reactive non-stress tests (NST) (25.53%) and abnormal Doppler findings were strongly associated with adverse outcomes, highlighting the need for close fetal monitoring and timely intervention.

Conclusion

Oligohydramnios is associated with a high rate of operative deliveries, increased neonatal morbidity, and adverse perinatal outcomes. Early identification and close fetal monitoring using Doppler studies and NSTs play a critical role in optimizing pregnancy outcomes. The findings reinforce the need for individualized management strategies to improve neonatal survival and reduce maternal complications.

## Introduction

Amniotic fluid plays a vital role in fetal development, acting as a protective cushion and facilitating musculoskeletal and pulmonary maturation. It is primarily derived from fetal urine and lung secretions, with absorption occurring through fetal swallowing and intramembranous pathways. Normal amniotic fluid levels ensure fetal well-being and prevent complications associated with cord compression and placental insufficiency [1]. Oligohydramnios is diagnosed when the amniotic fluid index (AFI) measures <5 cm or when the single deepest vertical pocket is <2 cm, as assessed by ultrasonography [2]. The condition occurs in 1-2% of pregnancies, increasing in post-term pregnancies, and is associated with an increased risk of intrauterine growth restriction (IUGR), umbilical cord compression, meconium aspiration, and non-reassuring fetal heart rate patterns [3].

The etiology of oligohydramnios varies and includes fetal, maternal, and placental factors. Common causes include congenital renal anomalies, preterm premature rupture of membranes (PPROM), uteroplacental insufficiency due to hypertension, diabetes, and other chronic maternal diseases. Medications such as non-steroidal anti-inflammatory drugs (NSAIDs) and angiotensin-converting enzyme (ACE) inhibitors have also been implicated in reducing amniotic fluid production [4].

Perinatal outcomes in oligohydramnios cases have been widely studied, with evidence suggesting increased neonatal complications, including low birth weight, preterm labor, and neonatal intensive care unit (NICU) admissions [5]. Oligohydramnios is also associated with higher cesarean section rates due to fetal distress, impacting maternal health outcomes [6].

Despite extensive research, controversy remains regarding the optimal management approach for oligohydramnios. Some studies advocate for expectant management with close fetal surveillance, while others recommend timely induction of labor to prevent adverse perinatal outcomes [7]. Understanding the maternal and fetal consequences of oligohydramnios is essential for guiding clinical decision-making and optimizing pregnancy outcomes.

Given the significant maternal and perinatal implications of oligohydramnios, this study aims to analyze maternal morbidity and perinatal outcomes in pregnancies between 28 and 42 weeks complicated by oligohydramnios. This research will provide valuable insights into risk stratification, management strategies, and potential interventions to improve outcomes in affected pregnancies.

## Materials and methods

This was a hospital-based cross-sectional study conducted over two years (October 2022 to September 2024) at Indira Gandhi Government Medical College and Hospital, a tertiary public healthcare referral and teaching hospital, in Nagpur, Maharashtra, India. The study population included pregnant women between 28 and 42 weeks of gestation diagnosed with oligohydramnios. The diagnosis was confirmed using ultrasound, with AFI <5 cm or a single deepest pocket <2 cm being the inclusion criteria [8].

The study was approved by Institute Ethics Committee, Board of Research Studies, Indira Gandhi Government Medical College and Hospital (approval number: IGGMC/Pharm/BORS/1256-57/2022). Written informed consent was taken from all the participants in their preferred vernacular language (Hindi/Marathi).

Inclusion and exclusion criteria

Consenting pregnant women aged 18-40 years with singleton pregnancies with gestational age ≥ 28 weeks of gestation, diagnosed oligohydramnios (AFI <5) with intact membranes, and in cephalic presentation formed the inclusion criteria. Exclusion criteria encompassed pregnancies with multiple gestations, fetal congenital anomalies, pregnancies complicated by polyhydramnios or pre-existing maternal renal disease, previous history of scarred uterus, and patients having concomitant antepartum haemorrhage or malpresentations.

Sample size calculation

The sample size was 188, calculated based on a prevalence rate of 40% (p=0.40) and an absolute error of 7%, with a significance level of 5%. [9,10] The formula used for calculation was:

$\frac{({Z_{1-\frac{\alpha}{2}}}{})\rho (1-\rho)}{\epsilon^{2}}$

Where ρ = 0.40, ϵ = absolute error = 7%, α = level of significance 5%, $Z_{1-\frac{\alpha}{2}}$ = 1.96 for a 95% confidence level (CI).

Amongst the antenatal clinic attendees, 188 pregnant women with oligohydramnios (AFI <5 cm) were included after meeting inclusion and exclusion criteria.

Data collection and parameters assessed

A pre-tested semi-structured proforma was used to collect demographic and clinical data, including maternal age, parity, and comorbidities, obstetric history and gestational age at diagnosis, AFI measurement and Doppler findings, mode of delivery (vaginal vs. cesarean), fetal distress and meconium-stained amniotic fluid, neonatal outcomes (Apgar scores, NICU admissions, birth weight, and perinatal mortality).

Ultrasound and Fetal Surveillance

Amniotic fluid was assessed ultrasonographically using the four-quadrant AFI method, which is widely accepted for diagnosing oligohydramnios [1]. Fetal well-being was monitored using non-stress tests (NST), Doppler velocimetry, and biophysical profiles. Expectant management involved hydration therapy and frequent monitoring, while cases with severe oligohydramnios necessitated expedited delivery.

Outcome Measures

After birth, neonatal Apgar scores were recorded at one and five minutes. Newborns with poor Apgar scores or requiring resuscitation were admitted to the neonatal intensive care unit (NICU) or ward, and the reasons for admission were documented. Maternal outcomes were assessed based on the mode of delivery and the need for labor induction. Perinatal outcomes were evaluated in terms of perinatal mortality (stillbirths and early neonatal deaths) and neonatal morbidity, including NICU admissions due to fetal distress, intrauterine growth retardation, or low Apgar scores. The incidence of congenital malformations was also noted.

Procedural details

Eligible participants were admitted to the obstetric ward and monitored until delivery. A detailed history, including personal, menstrual, and obstetric details, was recorded, followed by a thorough physical and systemic examination. Routine laboratory investigations, such as complete blood count (CBC), liver function test (LFT), and renal function test (RFT), were conducted at admission and repeated as required.

All women diagnosed with oligohydramnios underwent Doppler ultrasound to assess fetal well-being. Additionally, NSTs were performed biweekly to monitor fetal status. A reactive (normal) NST was defined as a baseline fetal heart rate (FHR) of 110-160 beats per minute, with at least two accelerations of ≥15 beats lasting at least 15 seconds within a 20-minute period, and no decelerations. For fetuses below 32 weeks, accelerations were defined as an increase in baseline FHR by 10 beats per minute for at least 10 seconds.

Management decisions were individualized, ensuring necessary treatment was provided. The mode of delivery was determined based on maternal and fetal conditions. Spontaneous labor onset was recorded.

Statistical analysis

The data collected were entered in Microsoft Excel version 2017 (Microsoft Corporation, Redmond, Washington, United States) and systematically analyzed using appropriate statistical methods to draw meaningful conclusions regarding the maternal and perinatal outcomes associated with oligohydramnios. Statistical analysis was performed using chi-square and t-tests to compare outcomes between oligohydramnios cases and normohydramnios controls. A p-value <0.05 was considered statistically significant.

## Results

The study included 188 pregnant women between 28 and 42 weeks of gestation diagnosed with oligohydramnios. Table 1 depicts the basic sociodemographic profile data of interest of the study participants. The mean maternal age in the study population was 25.4 ± 4.2 years, with the majority of participants (43.61%, n=82) in the age group of 21-25 years. Primigravida women constituted 61.70% (n=116) of the study population, while multigravida women comprised 38.30% (n=72).

**Table 1:** Sociodemographic distribution of study participants (N=188)

Characteristic	Distribution	Preterm Participants (< 37 weeks), n (%)	Term Participants (≥ 37 weeks), n (%)	Total, N (%)
Age (years)	≤ 20	10 (08.47)	15 (04.82)	25 (13.29)
	21–25	31 (16.48)	51 (27.13)	82 (43.61)
	26–30	19 (10.10)	38 (20.21)	57 (30.31)
	31–35	09 (04.79)	14 (07.44)	23 (12.23)
	36–40	01 (00.53)	00 (00.53)	01 (00.53)
	Total	70 (37.23)	118 (62.77)	188 (100)
Obstetric Score	Primigravida	42 (22.34)	74 (39.36)	116 (61.70)
	Multigravida	28 (14.90)	44 (23.40)	72 (38.30)
	Total	70 (37.23)	118 (62.77)	188 (100)

Table 2 summarizes the NST and Doppler findings of the study participants at the time of admission. Non-reactive NSTs were more common in preterm cases (47.1%, n=33) compared to term cases (12.7%, n=15), indicating a higher incidence of fetal compromise in preterm pregnancies. Abnormal Doppler findings were observed in 24.28% (n=17) of preterm pregnancies, compared to only 5.9% (n=7) of term pregnancies, suggesting a greater degree of uteroplacental insufficiency and fetal distress in early-onset oligohydramnios.

**Table 2:** Clinical parameters at admission in the study participants (N=188) LSCS: lower segment cesarian section

Gestational Age	Clinical Parameters	Vaginal delivery, n (%)	Assisted vaginal delivery (Vacuum), n (%)	Emergency LSCS, n (%)	Total, n (%)	P-values	Chi-Square Test
Non-Stress Test							
< 37 weeks (Preterm)	REACTIVE	9 (24.30)	0	28 (75.70)	37 (52.90)	0.76	0.09
	NON-REACTIVE	7 (21.20)	0	26 (78.80)	33 (47.10)		
	TOTAL	16 (22.90)	0	54 (77.10)	70 (100)		
≥ 37 weeks (Term)	REACTIVE	37 (35.90)	3 (2.90)	63 (61.20)	103 (87.30)	0.76	0.09
	NON-REACTIVE	2 (13.30)	0	13 (86.70)	15 (12.70)		
	TOTAL	39 (33.10)	3 (02.50)	76 (64.40)	118 (100)		
Doppler Changes on Ultrasound							
< 37 weeks (Preterm)	NORMAL	9 (12.86)	0	44 (62.86)	53 (75.71)	0.04	4.27
	ABNORMAL	7 (10.00)	0	10 (14.28)	17 (24.28)		
	TOTAL	16 (22.86)	0	54 (77.14)	70 (100)		
≥ 37 weeks (Term)	NORMAL	37 (33.33)	3 (02.54)	71 (64.00)	111 (94.10)	0.86	0.29
	ABNORMAL	2 (01.70)	0	5 (71.40)	7 (05.90)		
	TOTAL	39 (33.10)	3 (2.50)	76 (64.40)	118 (100)		

In terms of delivery outcomes, lower segment cesarean section (LSCS) was the predominant mode of delivery, accounting for 77.1% (n=54) of preterm cases and 64.4% (n=76) of term cases. Among term pregnancies, 33.1% (n=39) had vaginal deliveries, while 2.5% (n=3) required assisted vaginal deliveries using vacuum extraction. In preterm pregnancies, 22.86% (n=16) had vaginal deliveries, but none required assisted vaginal deliveries. Table 3 summarizes these findings. Among cesarean deliveries, the most common indications were fetal distress (38.2%, n=72), non-reassuring fetal heart rate patterns (21.4%, n=40), and failure to progress in labor (19.6%, n=37). In the patients who needed assisted vaginal delivery, the common indications included maternal exhaustion or prolonged second stage of labor.

**Table 3:** Perinatal outcomes and mode of delivery among the study participants (N=188) LSCS: lower segment cesarean section

Perinatal Outcome	Preterm (< 37 weeks), n (%)			Term (≥ 37 weeks), n (%)			Total, n (%)	P-value	Chi Square Test
	Vaginal Delivery	Assisted Vaginal Delivery	LSCS	Vaginal Delivery	Assisted Vaginal Delivery	LSCS			
Live birth	9 (04.79)	0	46 (24.47)	38 (20.21)	3 (1.60)	76 (40.42)	172 (91.50)	0.001	43.12
Early neonatal death (<7 days)	6 (03.19)	0	7 (3.72)	0	0	0	13 (6.91)		
Still born	1 (00.53)	0	1 (0.53)	1 (0.53)	0	0	3 (1.60)		
Total	16 (08.51)	0	54 (28.72)	39 (20.74)	3 (1.60)	76 (40.42)	188 (100)		

The study evaluated perinatal outcomes, including neonatal weight, Apgar scores, NICU admissions, and perinatal mortality. Low birth weight (<2.5 kg) was observed in 53.19% (n=100) of neonates, reflecting an increased risk of fetal growth restriction in pregnancies complicated by oligohydramnios. Apgar scores at birth indicated perinatal compromise, with 29.73% (n=55) of neonates having a score of ≤7 at one minute, requiring resuscitation in 7.4% (n=4) of cases. By five minutes, 13.29% (n=25) of neonates still had low Apgar scores, suggesting continued distress. NICU admissions were required in 14.36% (n=27/188) of cases, primarily due to respiratory distress syndrome (8.9%, n=5), meconium aspiration syndrome (5.7%, n=3.03), and neonatal sepsis (3.2%, n=6). Neonatal jaundice was observed in 11.6% (n=22) of cases, necessitating phototherapy in most instances. Table 4 summarizes these findings.

**Table 4:** Perinatal outcomes in the study participants (N=188) NICU: neonatal intensive care unit

Characteristics	Gestational Age, n (%)		Total, n (%)	P-value	Chi Square Test
	Term	Preterm			
APGAR SCORE AT 1 MINUTE					
< 7	17 (09.19)	38 (20.54)	55 (29.73)	<0.05	33.25
≥ 7	100 (54.0)	30 (16.21)	130 (70.27)		
Total	117 (63.24)	68 (36.76)	185 (100)		
APGAR SCORE AT 5 MINUTES					
< 7	3 (1.62)	22 (11.89)	25 (13.29)	< 0.05	30.15
≥ 7	114 (61.62)	46 (24.86)	150 (79.79)		
Total	117 (63.24)	68 (36.76)	185 (100)		
BIRTH WEIGHT					
Extremely Low Birth Weight (<1 Kg)	0	2 (01.06)	2 (01.06)		
Very Low Birth Weight (<1 – 1.5Kg)	0	7 (03.72)	7 (03.72)		
Low Birth Weight (1.5 - <2.5Kg)	37 (19.68)	54 (28.72)	91 (48.40)		
Average for Gestational Age (2.5Kg onwards)	81 (43.08)	7 (3.72)	88 (46.80)		
Total	118 (62.77)	70 (37.23)	188 (100)		
NEONATAL ADMISSION (BABIES HANDED WITH MOTHER = 123)					
Ward Admission	19 (30.64)	16 (25.81)	35 (56.45)	0.0015	45.5
NICU Admission	5 (8.06)	22 (35.49)	27 (43.55)		
Total	24 (38.71)	38 (61.29)	62 (100)		

Perinatal mortality was recorded at 8.51% (n=16), with 1.59% (n=3) of cases resulting in stillbirths and 6.91% (n=13) in early neonatal deaths, mainly attributed to severe birth asphyxia (1.8%, n=3), meconium aspiration syndrome (1.2%, n=2), and septicemia (0.8%, n=1). Meconium-stained amniotic fluid (MSAF) was observed in 14.9% (n=8) of cases, with 63.2% (n=5) of these neonates requiring immediate postnatal intervention. Fetal distress was present in 39.6% (n=75) of cases, as indicated by abnormal cardiotocographic (CTG) patterns and variable decelerations.

A sub-analysis comparing early-onset (28-34 weeks) and late-onset oligohydramnios (>34 weeks) revealed that preterm birth was significantly higher in the early-onset group (64.3%, n=45) compared to the late-onset group (9.8%, n=7). NICU admissions were also more frequent in early-onset cases (36.1%, n=10), primarily due to complications such as respiratory distress syndrome. Cesarean section rates were higher in the late-onset oligohydramnios group (71.2%, n=68), predominantly due to non-reassuring fetal heart rate patterns and failed labor induction. These findings underscore the importance of timely diagnosis and individualized management strategies to optimize perinatal outcomes in cases of oligohydramnios.

## Discussion

Oligohydramnios, defined as an AFI ≤ 5 cm, has been associated with significant maternal and perinatal morbidity. The findings of this study align with prior research demonstrating the increased incidence of fetal distress, low Apgar scores, and the necessity for operative deliveries in pregnancies complicated by oligohydramnios.

The study revealed a high rate of cesarean deliveries (LSCS) due to fetal distress, with an overall LSCS rate of 69.1%. This finding is consistent with studies by Jagatia et al., who reported an LSCS rate of 85.71% in oligohydramnios cases [9], and Bacchav and Waikar, who observed a 66% cesarean rate in a similar geographic region​ [10]. The higher operative delivery rate in oligohydramnios can be attributed to fetal heart rate abnormalities and meconium-stained amniotic fluid, leading to early intervention to reduce perinatal morbidity.

Non-reactive NSTs were observed in 25.53% of cases, with 86.7% of such cases resulting in LSCS, a figure comparable to that seen in Bhagat and Chawla's study (32.3%) [11]. This highlights the significance of NST in the antenatal evaluation of pregnancies with oligohydramnios. However, its specificity remains debatable, as indicated by Sultana et al., who reported that a low AFI alone is a poor predictor of adverse perinatal outcomes​ [12].

Perinatal outcomes in the present study were characterized by a perinatal mortality rate of 8.5%, with stillbirths and early neonatal deaths comprising 6.9% and 1.6%, respectively. These findings align with previous studies such as Casey et al. (6.4%) [3] and Golan et al. (6.3%)​ [7]. However, the mortality rate reported by Bansal and Deodhar (15%) was significantly higher, possibly due to variations in sample size and the availability of neonatal intensive care facilities [13].

Regarding neonatal morbidity, 33.51% of neonates required admission to the NICU or ward, primarily for respiratory distress syndrome (32.25%) and low birth weight (14.51%). A higher rate of neonatal admissions was observed in other studies, such as Chamberlain et al. (43%) [8] and Sonal and Sinha (49%)​ [14]. The lower rate in the current study could be attributed to improved intrapartum management and early interventions, including amnioinfusion.

A significant correlation was found between fetal Doppler abnormalities and operative deliveries. Among cases with abnormal Doppler findings, 68.8% of preterm and 71.4% of term neonates required LSCS. Hitschold et al. reported a similar LSCS rate of 71%, reinforcing the importance of Doppler assessment in predicting adverse outcomes​ [15].

Further, comparing the perinatal mortality between two studies in southeast Asia, the current study reported an 8.5% perinatal mortality rate, while Mohammed and Ahmed found a similar rate at 6.5% [16]. The leading causes of perinatal mortality in both studies included fetal hypoxia and birth asphyxia. Notably, Mohammed and Ahmed [16] identified fetal hypoxia as the most prevalent ultrasound finding, occurring in 21.5% of cases, whereas the current study emphasized abnormal Doppler findings in 24.28% of preterm cases. This highlights the role of Doppler ultrasound in predicting adverse fetal outcomes and guiding timely intervention.

Interestingly, the present study did not find a significant association between induction of labor and mode of delivery, unlike Casey et al., who reported an induction rate of 42% in oligohydramnios cases [3]. While induction remains a commonly employed strategy to manage oligohydramnios, its efficacy in reducing perinatal morbidity remains controversial.

The findings of this study reinforce the well-established association between oligohydramnios and adverse perinatal outcomes. The high rate of cesarean deliveries, increased NICU admissions, and perinatal mortality underscore the need for vigilant antenatal surveillance and timely intervention in pregnancies complicated by oligohydramnios.

Limitations and strengths

Despite these insights, the study has certain limitations. Being a single-center, hospital-based study, the findings may not be generalizable to a broader population, especially in different geographical or socioeconomic settings. The cross-sectional design restricts the ability to establish causal relationships between oligohydramnios and adverse perinatal outcomes. Also, the estimated power of the test for the sample size in the study was 27.8%. A larger sample size could have increased the strength of the study. Additionally, confounding factors such as maternal nutritional status and interobserver variability in ultrasonographic assessments were not extensively analyzed.

At the same time, the study has notable strengths. It provides a comprehensive evaluation of maternal and perinatal outcomes in oligohydramnios with a well-defined study population and standardized diagnostic criteria. The use of objective parameters, including Doppler findings and NST results, adds robustness to the study. Furthermore, the sample size of 188 cases enhances the reliability of the findings. These results contribute to the existing literature and offer valuable clinical insights for obstetricians managing pregnancies complicated by oligohydramnios.

## Conclusions

This study highlights the significant maternal and perinatal implications of oligohydramnios in pregnancies between 28 and 42 weeks of gestation. The findings reaffirm the strong association between reduced amniotic fluid levels and adverse pregnancy outcomes, including increased rates of cesarean section, fetal distress, low Apgar scores, and NICU admissions. The study also underscores the importance of antenatal surveillance, timely delivery, and individualized management strategies in mitigating risks associated with oligohydramnios. Early diagnosis and close fetal monitoring remain crucial for improving perinatal outcomes, particularly in cases with abnormal Doppler findings or non-reactive non-stress tests. Future research should focus on multicentric prospective studies with larger sample sizes to further validate these findings and explore potential interventions that could improve perinatal outcomes in oligohydramnios.

## Figures and Tables

**Figure 1:**
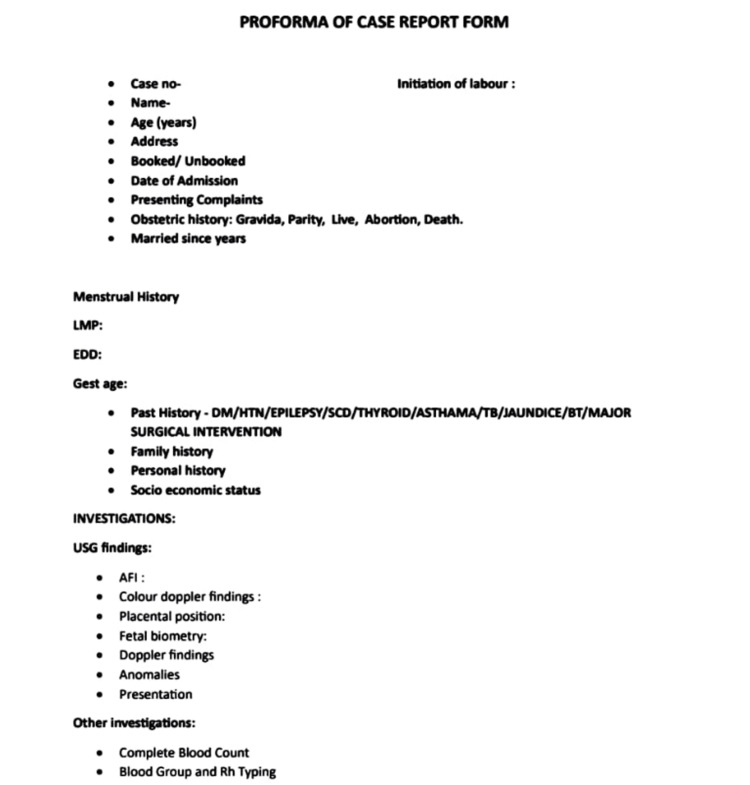
Page 1

**Figure 2:**
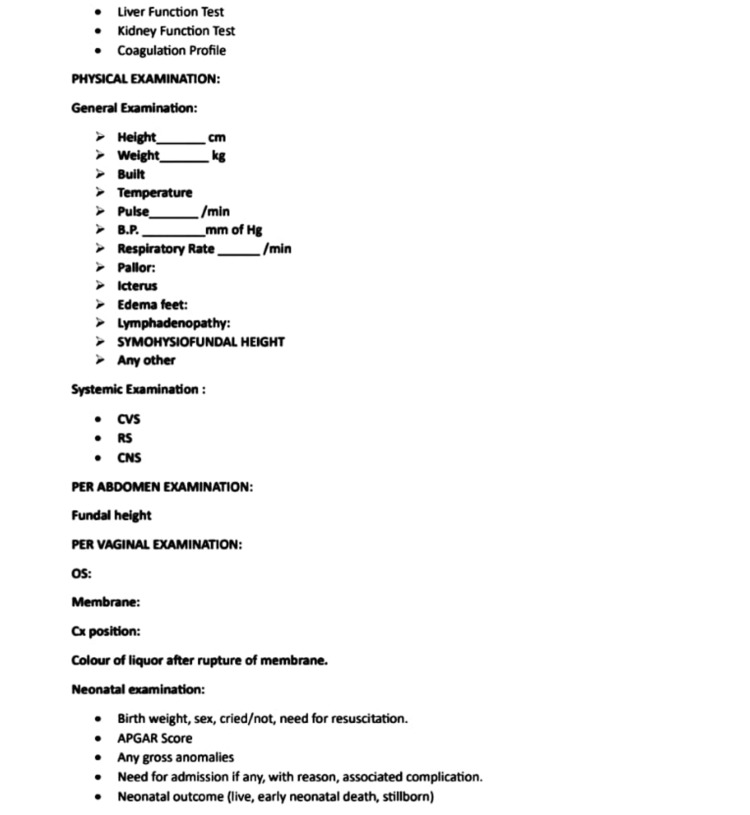
Page 2

## Appendices

Proforma
